# A Histone Acetyltransferase p300 Inhibitor C646 Induces Cell Cycle Arrest and Apoptosis Selectively in AML1-ETO-Positive AML Cells

**DOI:** 10.1371/journal.pone.0055481

**Published:** 2013-02-04

**Authors:** Xiao-ning Gao, Ji Lin, Qiao-yang Ning, Li Gao, Yu-shi Yao, Ji-hao Zhou, Yong-hui Li, Li-li Wang, Li Yu

**Affiliations:** 1 Department of Hematology, Chinese PLA General Hospital, Beijing, China; 2 Central Lab, Hainan Branch, Chinese PLA General Hospital, Sanya, China; Institut national de la santé et de la recherche médicale (INSERM), France

## Abstract

AML1-ETO fusion protein (AE) is generated by t(8;21)(q22;q22) chromosomal translocation, which is one of the most frequently observed structural abnormalities in acute myeloid leukemia (AML) and displays a pivotal role in leukemogenesis. The histone acetyltransferase p300 promotes self-renewal of leukemia cells by acetylating AE and facilitating its downstream gene expression as a transcriptional coactivator, suggesting that p300 may be a potential therapeutic target for AE-positive AML. However, the effects of p300 inhibitors on leukemia cells and the underlying mechanisms have not been extensively investigated. In the current study, we analyzed the anti-leukemia effects of C646, a selective and competitive p300 inhibitor, on AML cells. Results showed that C646 inhibited cellular proliferation, reduced colony formation, evoked partial cell cycle arrest in G1 phase, and induced apoptosis in AE-positive AML cell lines and primary blasts isolated from leukemic mice and AML patients. Nevertheless, no significant inhibitory effects were observed in granulocyte colony-stimulating factor-mobilized normal peripheral blood stem cells. Notably, AE-positive AML cells were more sensitive to lower C646 doses than AE-negative ones. And C646-induced growth inhibition on AE-positive AML cells was associated with reduced global histone H3 acetylation and declined *c-kit* and *bcl-2* levels. Therefore, C646 may be a potential candidate for treating AE-positive AML.

## Introduction

Leukemogenesis involves a variety of recurrent chromosomal abnormalities. t(8;21)(q22;q22) translocation is the most common chromosomal aberration identified in AML, which occurs in 40% of patients with French-American-British (FAB) M2 subtype and constitutes 12% of all newly-diagnosed cases [1]. This chromosomal translocation results in expression of AML1-ETO fusion oncogene. This oncogene encodes a fusion protein (AE) consisting of the conserved runt homology from hematopoietic transcription factor AML1 and the majority of ETO repressor, respectively encoded on chromosome 21 and 8. AE can repress gene expression via recruitment of co-repressors (e.g. NCoR and SMRT) and histone deacetylases by the ETO moiety [2]–[4], and it is also capable to activate gene expression [5]. Recently, it has been reported that AE binds the transcriptional coactivator p300 through its NHR1 domain, allowing AE and p300 to colocalize at the regulatory regions of various genes up-regulated by AE and involved in self-renewal of hematopoietic stem/progenitor cells (e.g. Id1, p21 and Egr1) [5]. The interaction between AE and p300 constitutes a key step for promoting self-renewal gene expression in leukemia cells and inhibition of p300 impairs its ability to promote leukemic transformation [5]. Therefore, p300 may be a potential therapeutic target for AE-positive leukemia.

p300 protein is a transcriptional co-activator with intrinsic histone acetyltransferase (HAT) activity, and it plays a crucial role in cell cycle progression, differentiation and apoptosis [6]–[9]. There is a distinct association between abnormal p300 activity and malignancies. Inhibition of p300 suppresses cellular growth in melanoma cells [10] and induces apoptosis in prostate cancer cells [11]. p300 activity is also required for G1/S transition in cancer cells [12]–[13]. Nevertheless, the fusion of the monocytic leukemia zinc finger protein gene to p300 gene has been identified in acute myeloid leukemia (AML) with t(8;22)(p11;q13) translocation, which is involved in leukemogenesis through aberrant histone acetylation [14]–[15]. The above evidence indicates the functional role of p300 as a tumor promoter and p300 inhibition may serve as a prospective approach for anti-tumor therapy.

Despite that anti-tumor activity of p300 inhibitors in other cancers has been reported [11], [16], its effects on leukemia cells and the underlying mechanisms have not been extensively investigated. C646, identified by using a structure-based in silico screening, is a competitive p300 inhibitor and more selective than other acetyltransferase [16]. C646 slows cell growth and impedes intracellular histone acetylation in several melanoma and lung cancer cell lines [16], prompting us to hypothesize that C646 might be a potential candidate for inhibiting cellular proliferation in AE-positive AML cells. Thus, we explored the effects of C646 on several AML cell lines, and primary blasts from a transgenic leukemia mouse model and initially-diagnosed AML patients. We found that C646 inhibited cellular proliferation, reduced colony formation, evoked partial cell cycle arrest in G1 phase, and induced apoptosis in AE-positive AML cells, while no significant inhibitory effects were observed in normal peripheral blood stem cells (PBSCs). Notably, the AE-positive AML cells were more sensitive to lower C646 doses than AE-negative ones. Moreover, C646-induced growth inhibition of AE-positive AML cells was associated with reduced histone H3 acetylation and declined *c-kit* and *bcl-2* levels. These results suggest a remarkable potential of C646 for treating AE-positive AML.

## Materials and Methods

### Animals and transplantation of leukemia cells

Female C57BL/6 mice (age 42.0±1.0 days, weight 16±0.2 g) were supplied by the experimental animal center of our hospital. A total of 1×10^6^ viable cryopreserved primary leukemia cells from AML1-ETO9a (AE9a) transgenic leukemia mice [17] (gifted by Shanghai Institute of Hematology, Shanghai, China) were injected into the tail vein of a C57BL/6 mouse. When the mouse became moribund, the spleen was separated under anesthesia for isolating fresh leukemia cells. After treated with C646 (Calbiochem, Darmstadt, Germany) or 0.1% DMSO for 24 h, the leukemia cells were injected to into the tail vein of 11 mice at a dose of 1×10^6^ viable cells/mouse. Animals were maintained in a room at 22–25°C under a constant day/night rhythm and given food and water *ad libitum*. All animal experiments were carried out in accordance with the National Institutes of Health Guide for Care and Use of Laboratory Animals and were approved by the Animal Care and Use Committee at our hospital.

### Clinical samples

The mononuclear cells from bone marrow samples with more than 70% blasts from 2 untreated AML patients, and granulocyte colony-stimulating factor-mobilized PBSCs from a healthy donor were obtained from Department of Hematology in our hospital, and prepared by Ficoll-Hypaque density gradient centrifugation (Sigma-Aldrich, St. Louis, USA). The FAB subtype and cytogenetic characteristics of the primary AML samples were as follows: patient #1, FAB M2, t(8;21)(q22;q22); patient #2, FAB M2, t(8;21)(q22;q22); patient #3, FAB M2, normal karyotype. This study was carried out in accordance with the principles of Declaration of Helsinki, and was approved by the Human Subject Ethics Committee in our hospital. Signed informed consent was obtained from each subject.

### Cell lines and cell cultures

Kasumi-1, SKNO-1, HL60, NB4, HEL, K562 and U937 cell lines were obtained from American Type Culture Collection (Rockville MD, USA). U937-AE cell line was a gift from Dr. Clara Nerv (University La Sapienza, Rome, Italy). This cell line was obtained by electroporation into U937 wild-type cells of an HA-tagged AE cDNA subcloned into a vector carrying the Zn^2+^-inducible mouse MT1 promoter [18]. U937-AE cell line was treated with 100 µM ZnSO_4_ for 8 h to induce AE expression.The above cell lines were maintained in RPMI-1640 medium (Invitrogen, Carlsbad, USA) supplemented with 10% fetal bovine serum. For SKNO-1 cells, 10 µg/L granulocyte-macrophage colony-stimulating factor (PeproTech, London, UK) was added to the medium. Primary AML blasts isolated from the spleen of transplanted AE9a leukemia mice or the bone marrow sample of AML patients, as well as normal PBSCs isolated from the healthy donor were cultured in Iscove's Modified Dulbecco's Medium (Invitrogen, Carlsbad, USA) supplemented with 20% fetal bovine serum, 100 µg/L stem cell factor, 10 µg/L interleukin-3, 10 µg/L interleukin-6 and 10 µg/L granulocyte-macrophage colony-stimulating factor (PeproTech, London, UK).

### Cell proliferation, cell cycle and apoptosis assays

Cells were seeded in 6-well plates at 1×10^6^ cells/well. C646 stocks (10 mM in anhydrous DMSO) were directly added to culture media at desired concentrations. DMSO concentration was kept constant at 0.1% among different treatments. After treatment with C646 or DMSO for 24 h, cells were harvested and subjected to the following assays. The number of viable cells was assessed by Cell Counting Kit-8 (Dojindo Laboratories, Kumamoto, Japan). For cell cycle assay, the cells were washed twice with ice cold PBS and fixed in 70% ethanol at 4°C overnight, followed by incubation with 10 µg/mL Ribonuclease A (Sigma-Aldrich, St Louis, MO) at 37°C for 30 min. The cells were then incubated with 50 µg/mL propidium iodide (BD Biosciences PharMingen, San Diego, USA). Flow cytometry analysis of DNA content was performed on a FACScalibur flow cytometer (Becton Dickinson, Franklin Lakes, NJ, USA). ModFit LT software (Version 3.1, Verity Software House Inc., Topsham, ME, USA) was used for subsequent analysis. For apoptosis assay, cells were stained with Annexin V-FITC (BD Biosciences PharMingen, San Diego, USA) or Annexin V-Alexa Fluor 647 (Life Technologies, Grand Island, USA), and analyzed by flow cytometer. FlowJo software (Version 7.6.1, Treestar, Ashland, OR, USA) was used for subsequent analysis.

### Colony formation assay

Cells were treated with C646 or DMSO as described above. After 24 h, cells were harvested and subjected to colony formation assay by using Methocult H4230 (STEMCELL Technologies Inc., Vancouver, Canada). For cell lines, they were plated in methylcellulose at a concentration of 1×10^3^ cells/mL. For primary leukemia cells, 100 µg/L stem cell factor, 10 µg/L interleukin-3, 10 µg/L interleukin-6 and 10 µg/L granulocyte-macrophage colony-stimulating factor were added to the methylcellulose medium and the cells were seeded at a concentration of 1×10^5^ cells/mL. Colony formation was assessed 7 to 14 d later The frequency of colony forming units (CFU) was calculated as number of colonies counted/number of cells plated.

### Western blot

Cells were treated with C646 or DMSO as described above. In some experiments, the pan-caspase inhibitor Q-VD-OPH (R&D Systems, Minneapolis, MN) was added at 50 µM 1 h prior to addition of C646. Total protein was extracted from cells using radio immunoprecipitation assay buffer (Sigma-Aldrich, St. Louis, USA) in the presence of proteinase inhibitor cocktail (Complete mini, Roche, Indianapolis, IN, USA). Polyacrylamide gel electrophoresis, tank-based transfer to Immobilon Hybond-C membranes (Amersham Biosciences) and immunodetection were performed with standard techniques. The following antibodies were used: caspase-9 (Asp330) antibody, cleaved caspase-8 (Asp391) (18C8) antibody, cleaved caspase-3 (Asp175) (5A1E) antibody (Cell Signaling Technology, Inc., Beverly, MA), AML1/RHD domain (Ab-2) antibody (Calbiochem, San Diego, CA), c-kit (C-19) antibody (Santa Cruz Biotechnology, Santa Cruz, USA), bcl-2 antibody (Bioworld Technology, St. Louis Park, USA), histone H3 antibody (Abcam plc., Cambridge, USA) and acetylated H3 antibody (Upstate Biotechnology, Buffalo, USA). β-actin antibody (Santa Cruz Biotechnology, Santa Cruz, USA) was used to normalize the amount of analyzed samples. Signals were visualized using Immobilon Western Chemiluminescent HRP substrate (Millipore Corporation, Billerica, MA) by exposure to films.

### Quantitative real-time PCR

RNA was isolated from cells using TRIzol reagent (Invitrogen, Carlsbad, USA) and cDNA was synthesized from 1 µg of total RNA using oligo(dT)_15_. Quantitative real-time PCR (qRT-PCR) was carried out in an ABI Prism 7500 Fast Real-time PCR System using TaqMan master mix (Applied Biosystems, Foster City, USA) according to the protocol. All data were normalized using the endogenous control (ABL). The sequences for the primers and probes were described in Table S1.

### Statistical analysis

SPSS 15.0 software (SPSS Inc., Chicago, IL) was used to process the data. Student's *t* test was applied to compare C646-induced changes to respective controls. The survival data were presented in a Kaplan-Meier format showing the percentage of mouse survival at various time-points post-transplantation. The overall survival comparisons among subtypes were performed by Mantel-Haenszel log-rank test. A *P* value of less than 0.05 was chosen as a threshold for statistical significance.

## Results

### C646 inhibited proliferation of Kasumi-1 and SKNO-1 cells through inducing cell cycle arrest and apoptosis

**Figure 1:**
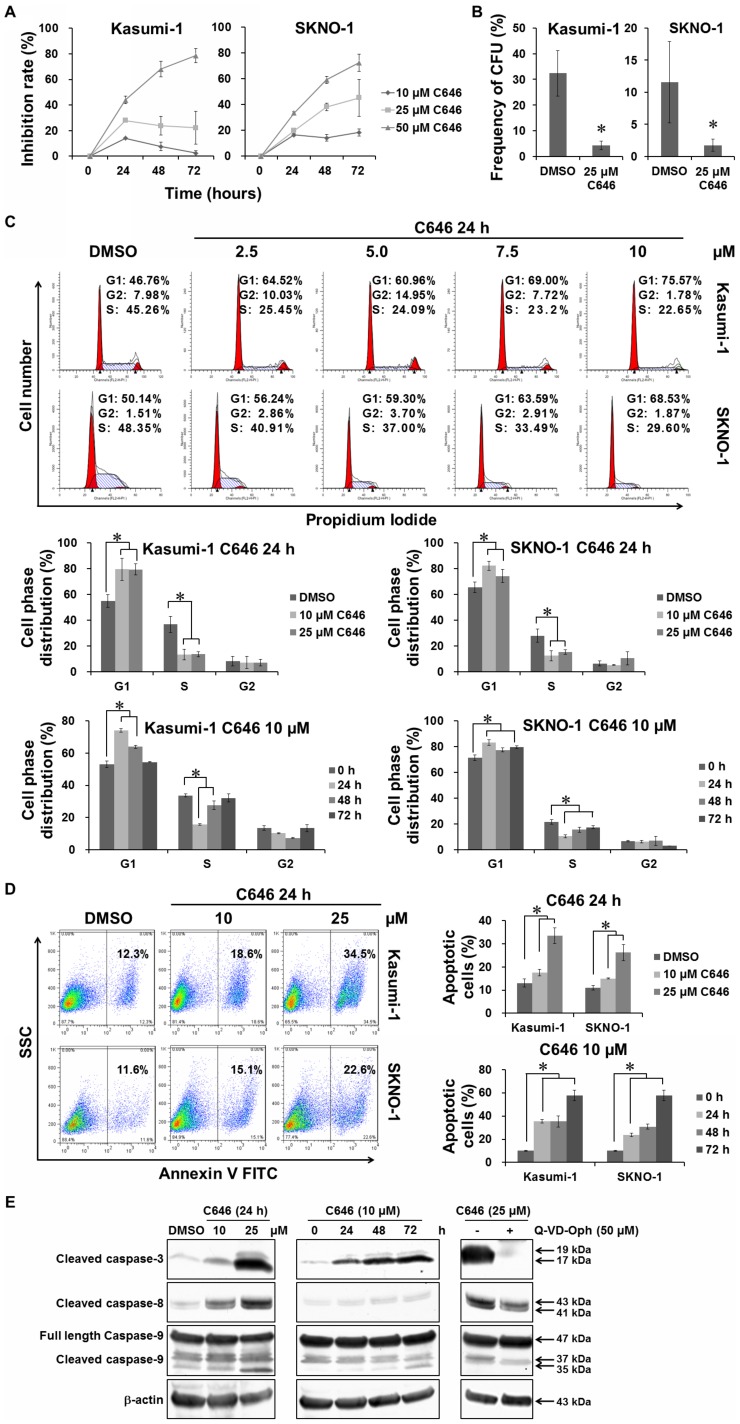
C646 inhibited proliferation of AE-positive AML cell lines through inducing cell cycle arrest and apoptosis. The cultured AE-positive AML cell lines Kasumi-1 and SKNO-1 cells were treated with given doses of C646 or 0.1% DMSO for 24 h before being subjected to the following assays. (A) C646-induced growth inhibition in both cell lines was detected by Cell Counting Kit-8 at the indicated times; means ± SD of 3 independent experiments. (B) C646-evoked ablation of leukemia colony-forming units in both cell lines was performed by colony formation assay. (C) Dose-dependent retardance of C646 on cell cycle distribution in both cell lines. The cells were stained with propidium iodide and measured by flow cytometry. (D) Dose- and time-dependent effects of C646 on apoptosis in both cell lines. The cells were stained with Annexin V-FITC and measured by flow cytometry. Histograms showed means ± SD of 3 independent experiments. * *P*<0.05. (E) C646 triggered caspase cleavage in Kasumi-1 cells. The Kasumi-1 cells treated with given doses of C646 in the presence or absence of 50 µM pan-caspase inhibitor Q-VD-OPH were collected and lysed at the indicated time points, and western blotting performed with the indicated antibodies. Equalization of protein loading was verified on the same membrane by reprobing after stripping. Data shown were representative of 2 independent experiments.

The AE-positive AML cell line Kasumi-1 has been proved more sensitive to C646 than the AE-negative AML cell line HL-60 and THP-1, as measured by growth inhibitory assay [5]. In the present study, we retested the growth inhibitory effects of C646 on Kasumi-1 and another AE-positive AML cell line SKNO-1, by using Cell Counting Kit-8 and methylcellulose colony formation assay. As shown in Figure 1A and 1B, both cellular growth and colony formation of Kasumi-1 and SKNO-1 cell lines were dramatically suppressed upon C646 treatment. We then investigated its effect on cell cycle via propidium iodide staining and flow cytometry. As shown in Figure 1C, C646 treatment for 24 h within the dose range from 2.5 to 10 µM induced a dose-dependent cell cycle arrest in G1 phase (upper pannel). However, when C646 dose was higher than 10 µM or the incubation time was longer than 24 h, its effect on cell cycle arrest did not increase accordingly (Figure 1C, lower pannel). Furthermore, C646 induced a dose-dependent upregulation in apoptosis determined by Annexin V-FITC staining and flow cytometry, with a minimum effective dose of 10 µM (Figure 1D). Although higher doses of C646 were required for inducing apoptosis than for inducing cell cycle arrest (10 *vs* 2.5 µM), the apoptotic percentage expressed a gradual increase over time. To further analyze the mechanism of C646-induced apoptosis, the dose- and time-course caspase cleavage were investigated by Western blot. C646 induced cleavage of caspases 3, 8, and 9 in Kasumi-1 cells. Increased cleavage occurred with increasing the concerntration or exposure time of C646 (Figure 1E). To determine whether inhibition of caspases would reduce caspase cleavage induced by C646, Kasumi-1 cells were incubated with 50 µM pan-caspase inhibitor Q-VD-OPH for 1 h, followed by treatment with 25 M C646 for 24 h. The C646-induced cleavage of caspases3, 8, and 9 was partially blocked by pretreatment with Q-VD-OPH (Figure 1E). These results indicated that the C646-evoked growth inhibition of AE-positive AML cell lines was associated with cell cycle arrest and induction of apoptosis.

### C646 was more selective to AE-positive AML cells than AE-negative cells

**Figure 2:**
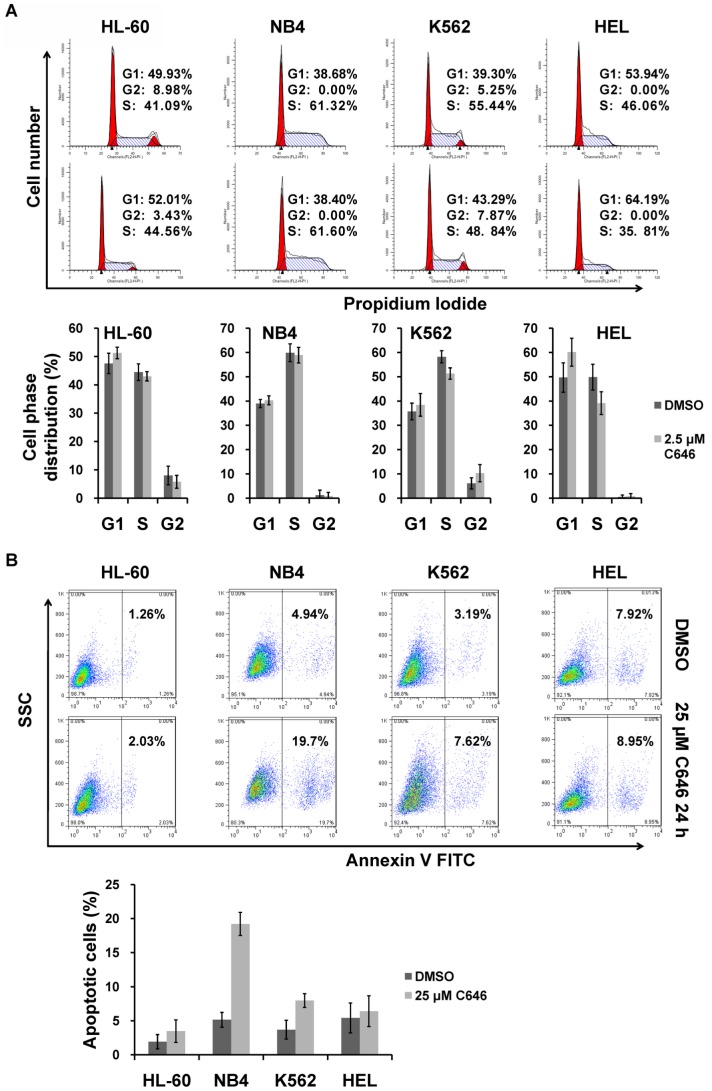
Effects of C646 on cell cycle distribution and apoptosis in AE-negative AML cell lines. Four AE-negative AML cell lines were respectively treated with given doses of C646 or 0.1% DMSO for 24 h before being subjected to the cell cycle distribution (A) and apoptosis (B) assays, as described in Figure 1. Histograms showed means ± SD of 3 independent experiments.

**Figure 3:**
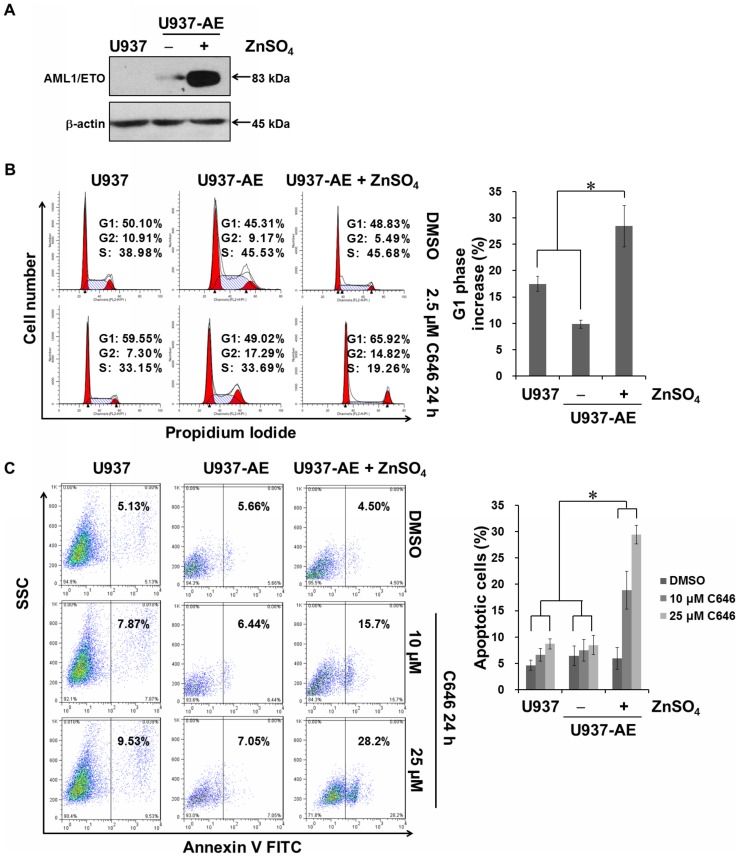
Selectivity of C646 for AE-positive AML cell lines. (A) AE expression in U937, U937-AE cell lines. U937-AE cells were treated in the absence or the presence of 100 µM ZnSO_4_ for 16 h. The cells were lysed and western blotting performed with the indicated antibodies. Equalization of protein loading was verified on the same membrane by reprobing after stripping. Data shown were representative of 2 independent experiments. Cells treated as in (A) were incubated further with given doses of C646 or 0.1% DMSO for 24 h before being subjected to the cell cycle distribution (B) and apoptosis (C) assays, as described in Figure 1. Histograms showed means ± SD of 3 independent experiments. * *P*<0.05.

We then tested whether C646 could induce cell cycle arrest or apoptosis in 4 AE-negative AML cell lines, HL-60, NB4, K562 and HEL. These cell lines treated with low dose of C646 (2.5 µM) showed only a marginal increase in the percentage of cells in G1 phase (Figure 2A). Even treated with 25 µM of C646, the apoptosis in HL-60, K562 and HEL cell lines could not be significantly triggered (Figure 2B). To further confirm the specificity of low dose of C646 for AE-positive cells, we evaluated its effects in an AE-inducible U937-AE cell line. The U937-AE cells grown in the presence of ZnSO_4_ with high expression of AE (Figure 3A), were more sensitive to the effects of C646 on cell cycle arrest and apoptosis than the cells grown in the absence of ZnSO_4_ and U937 wild type cells (Figure 3B and 3C). Together, these data suggested that C646 was more selective to AE-positive AML cells than AE-negative cells on inducing cell cycle arrest and apoptosis.

### C646 also inhibited the growth of primary AE-positive AML blasts

**Figure 4:**
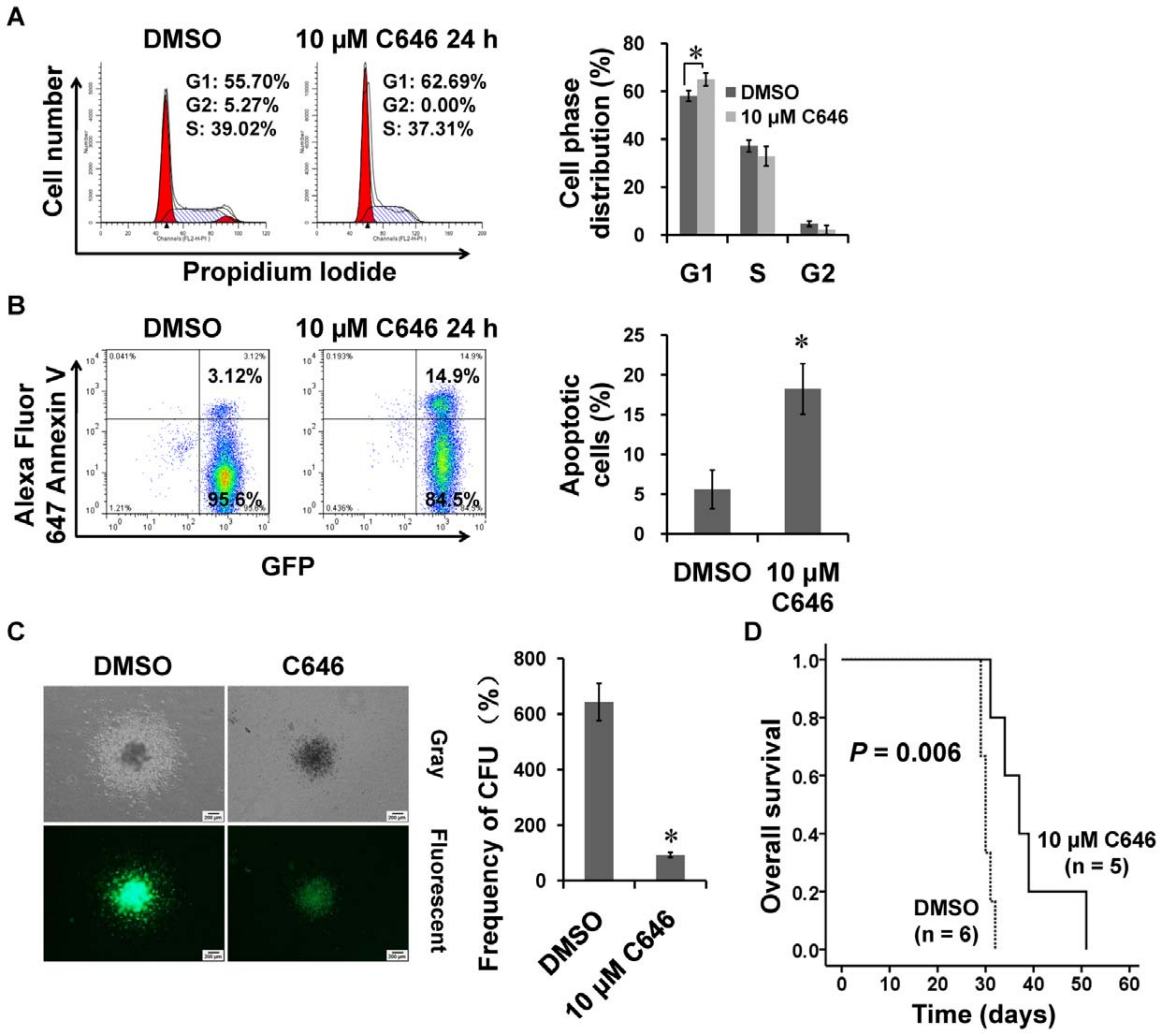
C646 inhibited *in vivo* proliferation of primary AML blasts isolated from AE9a leukemia mice. The AML blasts were isolated from the spleen of transplanted AE9a mice and cultured with 10 µM C646 or 0.1% DMSO for 24 h before being subjected to the cell cycle distribution (A), apoptosis (B) and colony formation (C) assays. Histograms showed means ± SD of 3 independent experiments. * *P*<0.05. (D) Primary AML blasts isolated from the spleen of transplanted AE9a leukemia mice were treated with C646 or DMSO and injected into the tail vein of C57BL/6J mice at a dose of 1×10^6^ cells/mouse, respectively, and the survival time of each mouse were recorded.

To address whether C646 had similar effects on primary AML blasts, we first assessed the effects of C646 on AE9a transgenic mice blasts. This mouse model harbors leukemia cells expressing the AE9a splice variant, which includes an extra exon (exon 9a) of the ETO gene, encodes a C-terminally truncated AE protein and is expressed in the majority of t(8;21) patients [18]. The AE9a fusion gene was coexpressed with enhanced green fluorescent protein (EGFP) in retroviral MigR1 vector. Therefore, we could monitor the leukemia blasts by detecting EGFP-positive cells via flow cytometry. As shown in Figure 4A and 4B, *in vitro* treatment of C646 induced a cell cycle arrest in G1 phase and a dramatic elevation in apoptotic percentage in the AML blasts isolated from the spleens of leukemia mice. The number and size of colonies formed *in vitro* were also markedly reduced upon C646 treatment (Figure 4C). Notably, the median survival time of recipient mice injected with C646-treated leukemia blasts were distinctly longer than those injected with DMSO-treated blasts (37 *vs* 30 d), indicating that C646 could suppress *in vivo* growth of transplanted leukemia blasts (Figure 4D).

**Figure 5:**
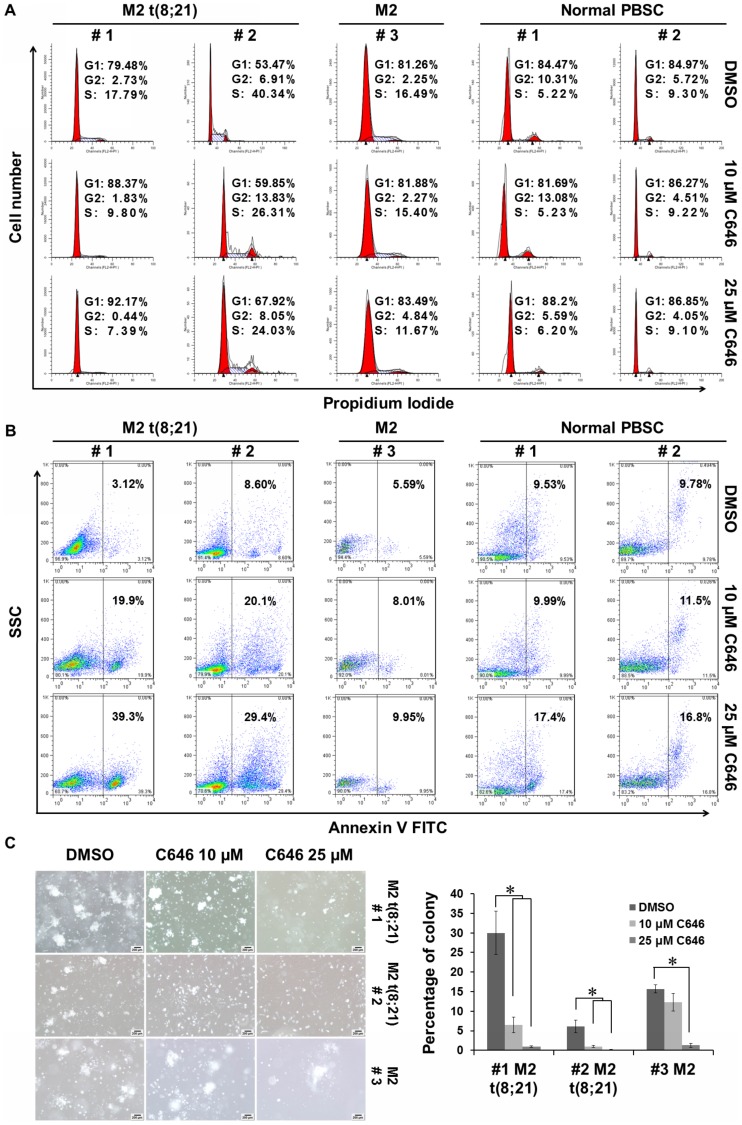
Selectivity of C646 for primary AE-positive AML blasts. The AML blasts were respectively isolated from the bone marrow samples of 2 t(8;21)(q22;q22) AML patients and a normal karyotype AML patient. The normal hematopoietic stem cells were isolated from granulocyte colony-stimulating factor-mobilized PBSCs of 2 healthy donors. The cells were cultured with given doses of C646 or 0.1% DMSO for 24 h before being subjected to the cell cycle distribution (A), apoptosis (B) or colony formation (C) assays. Histogram showed means ± SD for 2 independent experiments with triplicate cultures. * *P*<0.05.

Next, we assessed the effects of C646 on human primary leukemia blasts isolated from AE-positive and -negative AML patients and normal hematopoietic stem cells isolated from granulocyte colony-stimulating factor-mobilized PBSCs of 2 healthy donors. As shown in Figure 5A and 5B, C646 triggered marked cell cycle arrest and apoptosis in primary blasts from the AE-positive patients. The changes of cell cycle distribution and apoptosis observed in AE-negative sample and normal hematopoietic stem cells were considerably weaker compared with those in the AE-positive samples. Upon 10 µM of C646 treatment, a more significant reduction in colony formation was observed in AE-positive samples than that in AE-negative one, although the colony formation was strongly inhibited in both cases upon 25 µM of C646 treatment (Figure 5C). These results validated the high selectivity of C646 in the primary AE-positive AML blasts and its safety for normal hematopoietic stem cells.

### C646 reduced the levels of acetylated histone H3, c-kit and bcl-2 in Kasumi-1 and SKNO-1 cells

**Figure 6:**
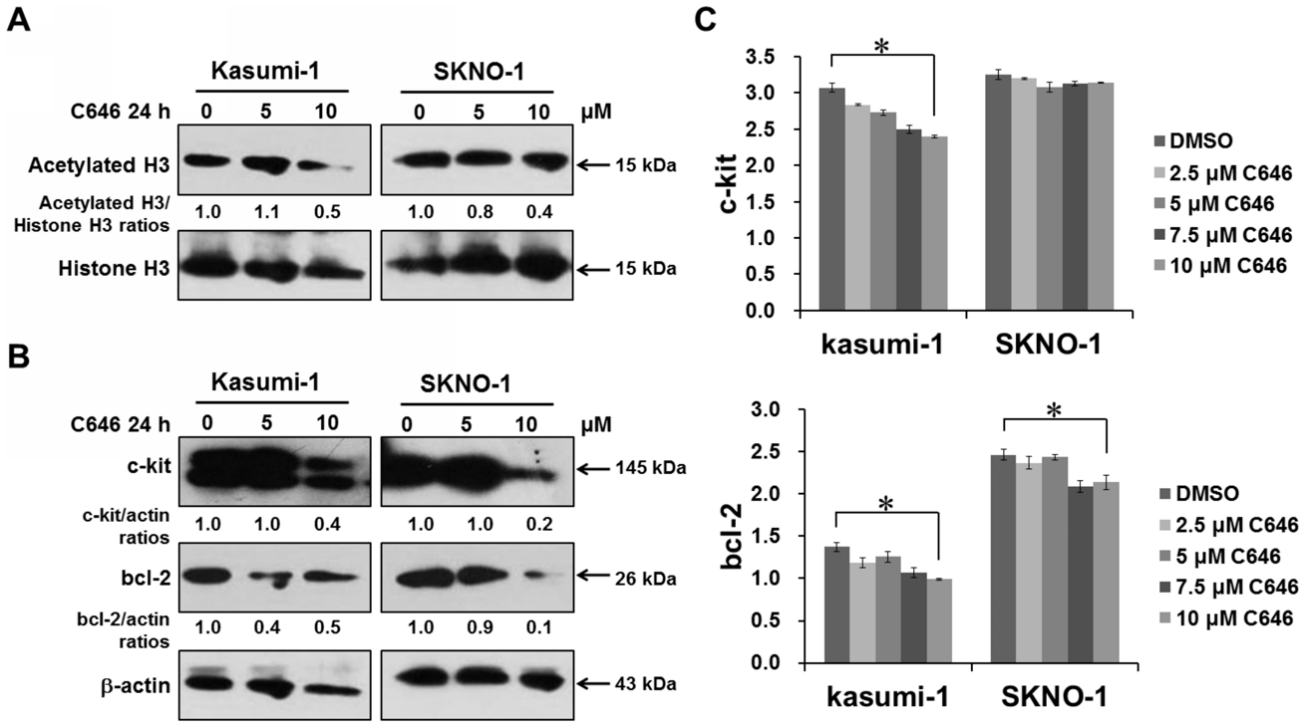
C646 reduced expression of acetylated histone H3, *c-kit* and *bcl-2* in AE-positive AML cell lines. Western blot analysis of (A) acetylated H3, total histone H3, (B) c-kit and bcl-2 proteins in Kasumi-1 and SKNO-1 cells after 24 h treatment with C646 or DMSO. The cells were lysed and western blotting performed with the indicated antibodies. Equalization of protein loading was verified on the same membrane by reprobing after stripping. Data shown were representative of 2 independent experiments. (C) qRT-PCR analysis of *c-kit* and *bcl-2* mRNA levels in the cells after 24 h treatment with C646 or DMSO. Histograms show relative mRNA levels normalized to control ABL gene; means ± SD of 3 independent experiments. * *P*<0.05.

To address the molecular mechanisms underlying C646-mediated cell cycle arrest and apoptosis, we detected the protein levels of acetylated H3 and total histone H3 in Kasumi-1 and SKNO-1 treated with and without C646. As expected, C646 treatment for 24 h induced significant reduction in global histone H3 acetylation in both cell lines (Figure 6A). Because *c-kit* proto-oncogene and *bcl-2* anti-apoptotic gene appear to be abnormal activation and closely related to apoptosis, cell cycle and proliferation in AE-positive AML cells [19]–[21], we detected the effects of C646 on protein and mRNA levels of *c-kit* and *bcl-2* by Western blot and qRT-PCR, respectively. Consistent with the induction of cell cycle arrest and apoptosis, a significant decrease of c-kit and bcl-2 protein levels were observed in Kasumi-1 and SKNO-1 cells treated with 10 µM of C646 (Figure 6B). There was also a slight decrease in *c-kit* and *bcl-2* mRNA levels in Kasumi-1 cells, as well as *bcl-2* mRNA levels in SKNO-1 cells, and no distinct alteration in *c-kit* mRNA levels in SKNO-1 cells (Figure 6C). These results revealed that post-transcriptional modulation such as C646-mediated histone deacetylation might participate in the repression of *c-kit* and *bcl-2* levels, which accounted for the growth-inhibitory activity of C646 in AE-positive AML cells.

## Discussion

In general, t(8;21) AML patients are considered as a favorable risk group, for more than 80% of younger cases can reach a complete remission. However, 40–50% of patients relapse and the long-term disease-free survival rate is around 60% [22]. Therefore, novel approaches to decrease the relapse of these patients are needed. As recruitment of HATs and histone deacetylases (HDACs) by AE fusion protein is a key step in AE-driven leukemogenesis, controlling HATs and HDACs may provide new targets for this subtype of leukemia. p300 belongs to a family of transcriptional coactivators with HAT activity, and C646 is a newly discovered competitive p300 inhibitor. C646 inhibits the growth of both melanoma and non-small cell lung cancer cell lines at 10 µM dose, with similar or higher potency as other p300 inhibitors [16]. C646 also inhibits the growth of primary blasts from t(8;21)-positive AML patients and Kasumi-1 cells, but has little effect on normal hematopoietic stem/progenitor cells [5]. Consistent with these reports, we also proved that C646 inhibited the growth and colony formation in AML cell lines Kasumi-1 and SKNO-1, which suggests a broad spectrum of anti-proliferation activity of C646 against tumor cell lines.

In addition to growth arrest, p300 is required for orderly G1/S transition in human cancer cells and inhibition of p300 induces block of progression into the S-phase of cell cycle and apoptosis [16], [23]. In our research, C646 succeeded in inducing cell cycle arrest in G1 phase and apoptosis specifically in AE-positive cells, while inappreciable effects were found in AE-negative cells. These data suggest the selectivity of C646 activity against AE-positive AML cells. The pan-caspase inhibitor Q-VD-OPH inhibited C646-induced cleavage of caspases 3, 8, and 9, confirming the caspase-dependent apoptotic process. This also suggests that both extrinsic and intrinsic pathways are triggered by C646, in keeping with recent findings which showed that the proapoptotic activity of C646 is determined via multiple apoptotic pathways [11]. In addition, it is noteworthy that neither cell cycle arrest nor apoptosis were observed in normal PBSCs on C646 treatment, which presents a valuable evidence for the drug safety of C646 in potential clinical uses. Although HDAC inhibitors have been applied in clinical trials both for solid and hematologic malignancies, there are limited reports about HAT inhibitors. Being a HAT inhibitor, C646 was proved very sensitive to primary blasts isolated from AE9a transgenic leukemia mice or an AE-positive AML patients in our study. These data further prompt the feasibility for C646 in pre-clinical application.

The ability of p300 to acetylate cellular proteins is critical for their functions in growth control. The HAT activity endows p300 the capacity to influence chromatin activity by modulating histones and several non-histone proteins [24]. To explore the underlying mechanisms of the apparent sensitivity of AE-positive leukemia cells to C646, we evaluated post-treatment levels of histone acetylation and expressions of *c-kit* and *bcl-2* in Kasumi-1 and SKNO-1 cell lines, which characterized by harboring *c-kit* mutation/overexpression, as well as *bcl-2* overexpression. Corresponding to the growth-inhibitory effects of C646 on AE-positive leukemia cells, there was a dose-dependent reduction in global histone H3 acetylation. It has been confirmed that aberrant activation of *c-kit* promotes cell cycle progression and contributes to abnormal cell proliferation by altering the tyrosine kinase signaling [19], and *c-kit* mutation cooperates with AE to induce leukemogenesis [20]. Moreover, acetylation of AE fusion protein by p300 participates in activating its targets [6], and AE can directly activate transcription of *bcl-2* [21]. It is reasonable to speculate that p300 also participates in AE-mediated transcriptional activation of *bcl-2*, and down-regulation of *c-kit* and *bcl-2* might involve in C646-mediated growth inhibition, cell cycle arrest and apoptosis in AE-positive AML cells. Therefore, the suppressive activity of C646 on aberrant expression of *c-kit* and *bcl-2* explains the high selectivity and sensitivity of AE-positive cells to C646. Certainly, the effects of C646 on AE-positive AML cells reflect a collective suppression of histone acetylation, *bcl-2*, *c-kit* and other factors. Identification of these uncharted factors and their roles in AML cells remains the subject of future investigations.

In conclusion, C646 exerts anti-leukemia effects on AE-positive AML cells. C646 inhibits cellular proliferation, reduces colony formation, evokes partial cell cycle arrest in G1 phase, and induces apoptosis in AE-positive AML cells, with reduced histone H3 acetylation and declined *c-kit* and *bcl-2* levels. The credible selectivity for AE-positive AML cells but not AE-negative ones, and the comparative safety for normal PBSCs provide C646 a nice perspective in the clinics. Further investigating the *in vivo* effects of C646 will undoubtedly promote its clinical application for relevant patients.

## Supporting Information

Table S1 **Sequences of the primers used in this study.**
(DOC)
