# Supplementary material for: A Histone Acetyltransferase p300 Inhibitor C646 Induces Cell Cycle Arrest and Apoptosis Selectively in AML1-ETO-Positive AML Cells
Source: PLoS One. 2013 Feb 4;8(2):e55481. doi: 10.1371/journal.pone.0055481 (PMC3563640; doi:10.1371/journal.pone.0055481)
Supplement: Table S1 — Sequences of the primers used in this study. (DOC) [file pone.0055481.s001.doc]

**Table S1.** Sequences of the primers used in this study

| Names |  |  | Sequences from 5' to 3' |
| --- | --- | --- | --- |
| c-kit | Sense: |  | GGGCCACCGTTTGGAAAG |
|  | Antisense: |  | TTACATTCAACCGTGCCATTG |
|  | *Probe:* |  | *FAM-TAGTGGTTCAGAGTTCTATAG-NFQ-MGB* |
| bcl-2 | Sense: |  | CGGTGGTGGAGGAGCTCTT |
|  | Antisense: |  | ACACATGACCCCACCGAACT |
|  | *Probe:* |  | *FAM-AGGATTGTGGCCTTCT-NFQ-MGB* |
| ABL | Sense: |  | CTCCATTATCCAGCCCCAAA |
|  | Antisense: |  | CCCAGCTTGTGCTTCATGGT |
|  | *Probe:* |  | *FAM-CGCAACAAGCCCACTG-NFQ-MGB* |
